# A Five-Species Transcriptome Array for Oral Mixed-Biofilm Studies

**DOI:** 10.1371/journal.pone.0027827

**Published:** 2011-12-14

**Authors:** Sylvio Redanz, Kerstin Standar, Andreas Podbielski, Bernd Kreikemeyer

**Affiliations:** Institute of Medical Microbiology, Virology and Hygiene, University Hospital Rostock, Rostock, Germany; Université d'Auvergne Clermont 1, France

## Abstract

**Background:**

Oral polymicrobial interactions and biofilm formation are associated with initiation and progression of caries, gingivitis, and periodontitis. Transcriptome studies of such interactions, allowing a first mechanistic insight, are hampered by current single-species array designs.

**Methodology/Principal Findings:**

In this study we used 385 K NimbleGene™ technology for design and evaluation of an array covering the full genomes of 5 important physiological-, cariogenic-, and periodontitis-associated microorganisms (*Streptococcus sanguinis*, *Streptococcus mutans*, *Fusobacterium nucleatum*, *Aggregatibacter actinomycetemcomitans*, and *Porphyromonas gingivalis*). Array hybridization was done with cDNA from cultures grown for 24 h anaerobically. Single species experiments identified cross-species hybridizing array probes. These probes could be neglected in a mixed-species experimental setting without the need to exclude the whole genes from the analysis. Between 69% and almost 99% of the genomes were actively transcribed under the mono-species planktonic, monolayer, and biofilm conditions. The influence of *Streptococcus mitis* (not represented on the array) on *S. mutans* gene transcription was determined as a test for a dual-species mixed biofilm setup. Phenotypically, under the influence of *S. mitis* an increase in *S. mutans* biofilm mass and a decrease in media pH-value were noticed, thereby confirming previously published data. Employing a stringent cut-off (2-fold, p<0.05), 19 *S. mutans* transcripts were identified with increased abundance, and 11 with decreased abundance compared to a *S. mutans* mono-species biofilm. Several of these genes have previously been found differentially regulated under general and acid stress, thereby confirming the value of this array.

**Conclusions/Significance:**

This new array allows transcriptome studies on multi-species oral biofilm interactions. It may become an important asset in future oral biofilm and inhibitor/therapy studies.

## Introduction

The oral cavity is a complex human habitat which is home to more than 750 different bacterial species [1]. Many of these bacteria are considered as members of the physiological flora living in symbiotic or commensal relationship to each other and their host. However, several bacterial species are pathogenic and have been reported as causative agents for oral diseases like caries, gingivitis, and periodontitis [1].

In most niches of the oral cavity physiological and pathogenic species live in large highly structured communities called biofilms [2]–[4]. Such biofilms develop after adherence of primary colonizing bacteria to inert or chemically conditioned surfaces. During biofilm maturation new bacterial species bind as secondary colonizers on top of the pioneer layers. Structure and integrity of biofilms is secured by production of macromolecular extracellular substances. Depending on the species composition an intense, inter- and intra-species communication occurs in such oral biofilms [5], [6]. Oral microbial biofilms may contain several hundred species and their composition can significantly differ among individuals [7], [8].

It is now appreciated that biofilm-involving oral diseases are among the most prevalent diseases worldwide, affecting nearly every human being at some time point during life span [9], [10]. Thus, investigation and comprehension of initiation and progression of these diseases, as well as developing novel strategies and therapies to control such biofilm-associated oral diseases is of great scientific interest and importance.

Numerous *in vitro* and *in vivo* studies have elucidated physiological and molecular details of bacterial interactions in oral biofilms of pathogenic and commensal bacteria. Such studies identified critical virulence factors of single species and allowed a first insight on the complex pathomechanisms acting in diseases like caries, gingivitis, and periodontitis [11]–[16]. It is now clear that mechanisms like cross-feeding and metabolic cooperation, as well as intra-/inter-species communication via quorum-sensing are critical for oral disease initiation and progression [5].

One level of influence in polymicrobial interactions is gene transcription. RT-qPCR and DNA-array design, based on full genome sequences, have boostered studies on expression profiles of oral bacteria. Specifically, single-species investigations employing typical stress forms encountered by the bacteria in their natural habitat were performed [17]–[19], planktonic versus biofilm bacterial growth was compared, and wild type and mutant strains were analyzed in such comparative transcriptomic approaches [20]–[22].

However, as the consortium of bacteria in the oral cavity is a multi-species association it would be an advantage and progress to analyze different oral bacteria with one array. Since to the best of our knowledge such a multi-species array was not previously available for transcriptome studies of oral bacteria, we developed a five-species custom array based on NimbleGen® DNA array platform technology. The 385 K™ chip design, allowing a total number of 385.000 gene probes per array, appeared to be a well suited platform to cover several bacterial genomes with up to three replicates per probe on each array. Our novel array design covered the full genomes of the cariogenic species *Streptococcus mutans* UA159, the physiological species *Streptococcus sanguinis* SK36, and the three periodontitis-associated species *Fusobacterium nucleatum* DSMZ 25586, *Porphyromonas gingivalis* W83, and *Aggregatibacter actinomycetemcomitans* HK1651. The chip contained 10.107 unique probe sets covering 10.186 genes. Most genes were covered by 13 individual probes.

Here we present transcriptome data of single-species cultures and biofilms, which allowed identification of cross-hybridizing single gene probes across the species represented on the array. As an example, the transcriptome of *S. mutans* under the influence of *S. mitis* was studied in a two-species biofilm. Array results were verified by Real time RT-qPCR.

This array is a powerful tool to investigate and understand the complexity of transcriptional changes in mixed-species biofilms and could be useful to study biofilm interfering substances and their mode of action.

## Results

### Single-species hybridizations on the five-species arrays allowed identification of cross-hybridizing gene probes

In advance of using this novel custom array design for multi-species experiments, it was mandatory to perform hybridizations with labeled cDNAs from single-species cultures and biofilms. Under the experimental setup described here, only *S. sanguinis* and *S. mutans* developed biofilm structures. All other tested species grew in the planktonic phase or dense monolayers in the culture flasks. A documentation of biofilm and monolayer formation of the single species is given in Supporting Figure S1.

Hybridization of labeled cDNA from a single species resulted in fluorescence signals which can be presented in three groups: i) the total number of fluorescence signals compared to total number of probes derived from the whole genome, ii) probes reacting with different fluorescence signal intensities, and iii) nonspecifically reacting probes derived from the other bacterial species.

The hybridization of labeled cDNA from 24 h *S. sanguinis* biofilms resulted in fluorescence signals for 9497 probes, representing 1550 transcriptionally active genes. Thus, 69.1% of the total number of 2244 genes is transcribed under the biofilm growth conditions used in this study. One gene was classified as highly transcribed (6 probes of this gene reached 75–100% of maximal fluorescence signal intensity of the array). Up to now this gene has been described to encode a *conserved hypothetical protein*. Further, among 38 probes belonging to 14 genes with fluorescence intensities of 50–75% above background signal, we found many chaperones: *dnaK* (SSA_2007), *dnaJ* (SSA_2005), *grpE* (SSA_2008), *clpL* (SSA_2096), responsible for protein folding and degradation, *ftsH* (SSA_0015), a Zn metallo-peptidase involved in cell division via sigma 32, *adcC* (SSA_0136) a putative Zn ABC transporter, and *rplN* (SSA_0117), a ribosomal protein (Supporting Table S1).

Besides, 58 probes were identified to give false positive signals. These probes were derived from 9 genes of *A. actinomycetemcomitans* HK1651, 5 genes of *S. mutans* UA159, 1 gene of *P. gingivalis* W83 and 1 gene of *F. nucleatum* ATCC 25586. Table 1 summarizes the numbers of probes and designations of corresponding genes, which were found to lead to nonspecific signals after single species hybridization of all bacterial species presented on the chip. More detailed information on these probes and genes is presented in Supporting Table S2. Figure S1a shows the signal intensities graphically. However, since most genes were covered by up to 13 probes on the array there is only in rare cases a need to fully exclude complete genes from the analysis. For example, *S. sanguinis* cDNA cross-hybridized with the gene probe AA00182P0047 from *A. actinomycetemcomitans* HK1651 (see Supporting Table S2). Supporting Table S3 revealed that the gene AA00182 is covered by 13 individual gene probes. Thus, 12 non-crossreactive gene probes covering AA00182 are still available for evaluation of differential expression in an *A. actinomycetemcomitans* HK1651 - *S. sanguinis* mixed-species setting (Table 2).

**Table 1:** The number of cross-hybridizing probes.

	cross-reaction with:				
hybridization with:	*S. mutans*	*S. sanguinis*	*P. gingivalis*	*F. nucleatum*	*A. actinom.*
***S. mutans***	**24210 probes**	187 probes	16 probes	11 probes	68 probes
(1927 genes)	**(1915 genes)**	(67 genes)	(16 genes)	(11 genes)	(17 genes)
***S. sanguinis***	14 probes	**9497 probes**	1 probe	1 probe	42 probes
(2244 genes)	(5 genes)	**(1550 genes)**	(1 gene)	(1 gene)	(9 genes)
***P. gingivalis***	8 probes	12 probes	**21355 probes**	4 probes	50 probes
(1883 genes)	(2 genes)	(5 genes)	**(1771 genes)**	(4 genes)	(13 genes)
***F. nucleatum***	5 probes	22 probes	3 probes	**5223 probes**	24 probes
(1964 genes)	(4 genes)	(15 genes)	(3 genes)	**(1614 genes)**	(12 genes)
***A. actinom.***	9 probes	12 probes	8 probes	7 probes	**6702 probes**
(2168 genes)	(3 genes)	(5 genes)	(3 genes)	(4 genes)	**(1165 genes)**

Identification of none-specific hybridizing probes after single hybridization of *S. mutans* UA159, *S. sanguinis* SK36, *P. gingivalis* W83, *F. nucleatum* ATCC25586 and *A. actinomycetemcomitans* HK1651.

The table shows cross-hybridizing probes affecting different numbers of genes (in brackets) after single-species hybridization. Results which refer to the species hybridized on the chip are displayed in bold characters.

**Table 2:** Exemplary genes including cross-hybridizing probes.

Gene ID	Probe ID					
AA00182	AA00182P00018	AA00182P00311	**AA00182P00470^1^**	AA00182P00623	AA00182P00654	AA00182P00739
AA00182	AA00182P00018	AA00182P00311	**AA00182P00470^2^**	AA00182P00623	AA00182P00654	AA00182P00739
AA00182	AA00182P00018	AA00182P00311	**AA00182P00470^3^**	AA00182P00623	AA00182P00654	AA00182P00739
SSA_0123	**SSA_0123P00096^2^**	**SSA_0123P00101^2^**	**SSA_0123P00105^2^**	**SSA_0123P00110^2^**	**SSA_0123P00115^2^**	**SSA_0123P00126^2^**
SSA_0440	**SSA_0440P00036^2^**	**SSA_0440P00039^2^**	**SSA_0440P00042^2^**	**SSA_0440P00046^2^**	**SSA_0440P00049^2^**	**SSA_0440P00051^2^**
SSA_2391	**SSA_2391P00012^2^**	**SSA_2391P00013^2^**	**SSA_2391P00014^2^**	**SSA_2391P00015^2^**	**SSA_2391P00016^2^**	**SSA_2391P00017^2^**

Probe ID						
AA00182P00851	AA00182P01106	AA00182P01117	AA00182P01129	AA00182P01135	AA00182P01141	AA00182P01177
AA00182P00851	AA00182P01106	AA00182P01117	AA00182P01129	AA00182P01135	AA00182P01141	AA00182P01177
AA00182P00851	AA00182P01106	AA00182P01117	AA00182P01129	AA00182P01135	AA00182P01141	AA00182P01177
**SSA_0123P00222^2^**	**SSA_0123P00236^2^**	**SSA_0123P00237^2^**	**SSA_0123P00238^2^**	**SSA_0123P00240^2^**	**SSA_0123P00243^2^**	**SSA_0123P00296^2^**
**SSA_0440P00053^2^**	**SSA_0440P00174^2^**	**SSA_0440P00177^2^**	**SSA_0440P00178^2^**	**SSA_0440P00179^2^**	**SSA_0440P00180^2^**	**SSA_0440P00181^2^**

Exemplary genes and their probes represented on the described array. Cross-hybridizing probes are indicated by bold characters.

Footnotes indicate probes which were positive after hybridization with cDNA from *P.gingivalis* (^1^), *S. mutans* (^2^) and *S. sanguinis* (^3^), respectively.

Chip-hybridization with cDNA from *S. mutans* UA159 24 h biofilms revealed 99.4% of the genome actively transcribed under the experimental conditions described here. 24.210 probes of *S. mutans* UA159, representing 1915 genes from a total number of 1927 genes, were detected. Among the highly abundant transcripts (438 probes covering 167 genes with 75–100% of the maximal fluorescence signal intensity of the array), we identified also chaperones like *dnaK* (SMU_82), *dnaJ* (SMU_83), and *groEL* (SMU_1954). Further, *ftsZ* (SMU_552), a putative cell division protein, *dnaI* (SMU_1921), coding for a DNA helicase, *pflA*(SMU_1692), coding for pyruvat formiat lyase A, and several genes encoding ribosomal proteins like S7 (SMU_358), L27 (SMU_849), S1 (SMU_1200), S11 (SMU_2002) were found (Supporting Table S1). A total of 111 genes from the other species on the chip were found to react nonspecifically (refer to Table 1, Supporting Table S2 and Supporting Figure S2b). Again, with the information from Supporting Table S2 (number and identity of individual cross-hybridizing probes per gene) and the information from Supporting Table S3 (total number of individual probes per gene on the array) the experimentalist can decide if whole genes need to be excluded from two-species analysis.

The hybridization with labeled cDNA of *P. gingivalis* W83 cultures revealed measurable fluorescence intensities for 21855 probes of *P. gingivalis* representing 1771 genes of 1883 genes of this strain (94.1% of the genome). Among them, 208 highly transcribed genes were found, represented by 888 probes with 75–100% of the maximal fluorescence signal intensity of the array. These genes encoded chaperones like *dnaK* (PG_1208), *groES* (PG_0521), *groEL* (PG_0521), *clpL* (PG_0010), *clpX* (PG_0417) and several ribosomal proteins, like L27 (PG_0315), S9 (PG_376), L1 (PG_391), and S6 (PG_595) (Supporting Table S1). Only 75 probes originating from 24 genes of the other species reacted nonspecifically (refer to Table 1, Supporting Table S2 and Supporting Figure S2c). Apparently, the designed probe sets for *P. gingivalis* W83 are highly species -specific.

For *F. nucleatum* DSMZ 25586, which grew in dense monolayers after 24 h incubation, 25586 fluorescence signals were obtained for 5223 probes belonging to 1614 of 1964 genes of this species (82.2% of the genome). Only 7 genes, represented by 7 probes, showed very high fluorescence intensities, suggesting only a small number of genes as highly transcribed under the chosen conditions (Supporting Table S1). Among them, we identified genes encoding for a malonyl-CoA transacylase (FN0149), acetyl-CoA acetyltransferase (FN0495), a alkyl hydroperoxide reductase (FN1983) and also *ftsZ* (FN1451) involved in cell division. Further, 12 genes of *A. actinomycetemcomitans* HK1651, 15 genes of *S. sanguinis* SK36, 4 genes of *S. mutans* UA159 and 3 genes of *P. gingivalis* W83 displayed nonspecific reactivities (refer to Table 1, Supporting Table S2 and Supporting Figure S2d).

Hybridization of *A. actinomycetemcomitans* labeled cDNA resulted in fluorescence signals for 6702 probes of *A. actinomycetemcomitans*, representing 1165 of 2138 genes of this species. Particularly, 8 genes, represented by 54 probes, were found to be highly transcribed (75–100% of the maximal fluorescence signal intensity of the array). Except for *ccmB* (AA00059), a gene encoding for a heme exporter protein, all other genes have unknown or hypothetical functions (Supporting Table S1). 36 probes led to nonspecific signals potentially affecting the analysis of 15 genes of the other species presented on the chip (refer to Table 1, Supporting Table S2 and Supporting Figure S2e).

Taking into account the relatively low number of nonspecific cross-species hybridization signals identified in this series of experiments, the initial sequence analysis and algorithms for probe design proved to be of high quality. In only 3 cases, all probes derived from one gene showed nonspecific signals and thus, would require the exclusion of these genes from further analysis in the respective two-species setting (listed in Table 2). After performing these corrections, the combination of cDNAs from two or more species of the array should lead to truly positive signals in all possible combinations.

### Differential transcriptome of *S. mutans* in a dual-species biofilm with *S. mitis*

After testing the array performance in single-species transcriptome studies we next analyzed a dual-species model. A previous study from our lab has documented that the cariogenic species *S. mutans* grows to significantly increased biofilm mass in a dual culture with the physiological species *S. mitis* over a five day investigation period [23]. At the time of array design no full *S. mitis* genome sequence was available, thus this species is not covered by the array. However, the taxonomic relationship between both species could interfere with the suitability of any array to analyze the specific transcriptome of the two species. Thus, with the next series of experiments we addressed several issues: i) was the design of the present array suitable to discriminate the transcriptomes of two closely related species, ii) could this issue be tackled in absence of the genome sequence of one species, and iii) would the array hybridization experiments render results which could explain the observed behavior of *S. mutans* mixed-species biofilms? Therefore, we first tested potential cross-reactions of *S. mitis* cDNA with the 5 species genome array by exclusively using *S. mitis* cDNA. In this assay, 311 *S. mutans* probes displayed nonspecific reactions (Supporting Table S4). These nonspecifically reacting probes, but not the whole genes, were excluded from the data analysis of the *S. mutans*/*S. mitis* single-species/co-culture experiments.

Under the chosen conditions, *S. mutans* was able to form large biofilm masses already after 24 hours of incubation. The combination of *S. mutans* with *S. mitis* resulted in larger biofilm masses than obtained for the *S. mutans* mono-species culture. Culture supernatant pH-values of the combination were lower than for the *S. mutans* mono-species culture (4.96 versus 5.8 after 24 hours) but numbers of colony forming units of *S. mutans* were unchanged within the boundaries of experimental error over a five day co-culture period with *S. mitis* [23]. In contrast, *S. mitis* could be detected via different microscopic techniques, but no colony forming units were found.

**Figure 1:**
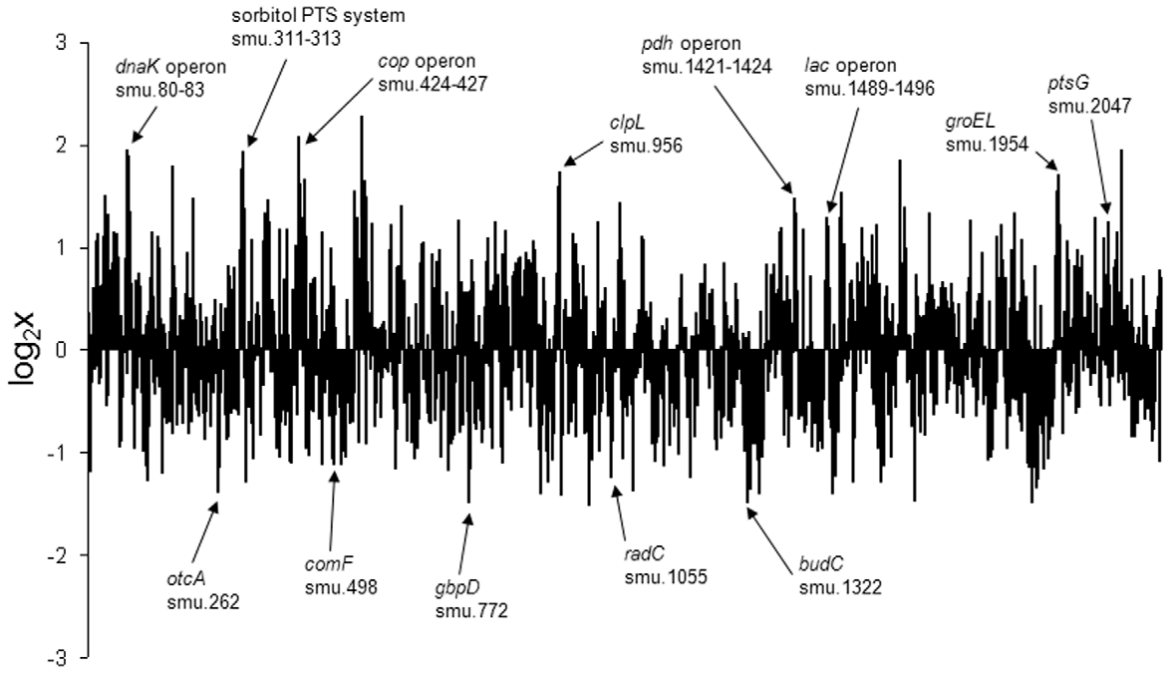
Overview of transcript level changes of *S. mutans* in co-culture with *S. mitis* compared to its single-species culture. Shown are the fold changes of all *S. mutans* genes represented on the array averaged from three independent replicates. Marked are up- or down- regulated genes and operons with a fold change of >2 including one or more genes significantly up- or down-regulated (p<0.05) except *clpL* (p = 0.069) and *groEL* (p = 0.075).

After deletion of all nonspecifically reacting probes from further analysis, the *S. mutans* transcriptome after co-culture with *S. mitis* showed a significantly different transcript abundance for 30 genes (fold change two, p<0.05) (19 were found up-regulated, 11 were down-regulated) compared to the *S. mutans* mono-species biofilm transcriptome. Using the Internet platform KEGG (Kyoto Encyclopedia of Genes and Genomes, http://www.genome.jp/kegg/), we found that the majority of these differentially regulated genes were involved in metabolism and environmental information processing (membrane transport) (Table 3). Of note, the stress response operon *dnaK* (smu_80-83) and the *cop*-operon (SMU_424-smu_427), as well as *groEL* (SMU_1954) and *clpL* (SMU_956), which have been described to be involved in stress response, showed high transcript abundances (Figure 1). Moreover, the agmatine deiminase gene cluster [24] (SMU_262-264) was found downregulated.

**Table 3:** Summary of the differential transcript abundances after transcriptome analysis.

Class/Gene ID	Gene name/Description	Fold change^a^	Direction ofregulation
**Metabolism**			
SMU_1140c	conserved hypothetical protein	2.11	down
SMU_1322	budC - putative acetoin dehydrogenase	2.78	down
SMU_1421	pdhC - putative dihydrolipoamide acetyltransferase, E2 component	2.78	up
SMU_1422	pdhB - putative pyruvate dehydrogenase E1 component subunit beta	2.32	up
SMU_1424	pdhD - putative dihydrolipoamide dehydrogenase	2.54	up
SMU_1493	lacD - tagatose-1,6-bisphosphate aldolase	2.47	up
SMU_262	otcA/aguB – putative putrescine carbamoyltransferase	2.07	down
SMU_264	aguA – agmatine deiminase	2.6	down
SMU_318	putative hippurate hydrolase	2.62	up
SMU_85	thiD - putative phosphomethylpyrimidine kinase	2.55	up
**Environmental Information Processing**			
SMU_1934c	putative cobalt ABC transporter, ATP-binding protein	2.07	down
**Metabolism, EnvironmentalInformation Processing**			
SMU_2047	ptsG - putative PTS system, glucose-specific IIABC component	2.13	up
SMU_311	PTS system, sorbitol (glucitol) phosphotransferase enzyme IIC2	3.41	up
SMU_312	PTS system, sorbitol phosphotransferase enzyme IIBC	2.93	up
SMU_313	putative PTS system, sorbitol-specific enzyme IIA	3.86	up
**Genetic information processing**			
SMU_1055	radC - putative DNA repair protein RadC	2.35	down
SMU_387	putative glycoprotein endopeptidase	2.06	down
SMU_424	copY - negative transcriptional regulator, CopY	4.25	up
SMU_427	copZ - putative copper chaperone	3.74	up
SMU_772	gbpD - glucan-binding protein D with lipase activity; BglB-like protein	2.8	down
**Genetic information processing,Environmental Information Processing**			
SMU_498	comF - putative late competence protein	2.16	down
SMU_81	grpE - heat shock protein GrpE (HSP-70 cofactor)	2.97	up
**Others**			
SMU_314	hypothetical protein	3.58	up
SMU_36	conserved hypothetical protein	2.84	up
SMU_428	conserved hypothetical protein	3.09	up
SMU_497c	conserved hypothetical protein	2.08	down
SMU_52	conserved hypothetical protein	2.21	up
SMU_560c	conserved hypothetical protein	2.5	up
SMU_916c	conserved hypothetical protein	2.64	down
SMU_982	bglB2 - putative BglB fragment	2.19	up

Displayed are differentially regulated genes of *S. mutans* after co-cultivation with *S. mitis* in biofilm structures compared to the *S. mutans* mono-species biofilm. Clustering was performed with the help of KEGG (Kyoto Encyclopedia of Genes and Genomes, http://www.genome.jp/kegg/).

a n-fold change after gene expression array analysis of three biological replicates (p<0.05).

The microarray data have been deposited in the Gene Expression Omnibus (GEO) database under accession number GSE28841.

### Confirmation of transcriptome results by Real Time RT-qPCR

As an independent second method we used RT-qPCR for validation of the observed transcriptome data. A variety of genes showing high or low transcript abundances in transcriptome analysis were chosen for further analysis with cDNA from a new series of biologically independent experiments. Precisely, 7 up-regulated and 6 down-regulated genes were selected. The gene SMU_2015 was used as control as it showed no change of expression (p>0.95) in the array experiments.

Table 4 shows the results of RT-qPCR as well as the comparison to the data of transcriptome analysis. In general, RT-qPCR confirmed the results of transcriptome experiments.

**Table 4:** Verification of array results by RT-qPCR analysis of selected genes.

Gene ID	Gene name/Description	Fold changeof RT-PCR^a^	Fold change oftranscriptome analysis^b^
**upregulated genes**			
SMU_81	*grpE*; heat shock protein GrpE (HSP-70 cofactor)	2.04 (0.51)	2.96 (p<0.05)
SMU_312	PTS system, sorbitol phosphotransferase enzyme IIBC	6.12 (1.25)	2.92 (p<0.05)
SMU_426	*copA*; copper-transporting ATPase; P-type ATPase	3.25 (0.83)	3.31 (p = 0.072)
SMU_956	*clpL*; putative Clp-like ATP-dependent protease, ATP-binding subunit	5.11 (3.3)	3.87 (p = 0.069)
SMU_1495	*lacB*; galactose-6-phosphate isomerase, subunit LacB	2.09 (0.52)	2.03 (p = 0.15)
SMU_1954	*groEL*; putative chaperonin GroEL	2.15 (0.83)	2.33 (p = 0.075)
SMU_2047	*ptsG*; putative PTS system, glucose-specific IIABC component	3.88 (2.79)	2.13 (p<0.05)
**downregulated genes**			
SMU_262	*otcA*; putative ornithine carbamoyltransferase	−2.39 (1.55)	−2.07 (p<0.05)
SMU_498	*comF*; putative late competence protein	−2.03 (0.93)	−2.16 (p<0.05)
SMU_772	*gbpD*; glucan-binding protein D with lipase activity; BglB-like protein	−3.06 (0.06)	−2.8 (p<0.05)
SMU_1055	*radC*; putative DNA repair protein RadC	−1.64 (0.45)	−2.35 (p<0.05)
SMU_1340	*bacA2*; putative surfactin synthetase	−3.36 (0.39)	−1.87 (p = 0.054)
SMU_1934c	putative cobalt ABC transporter, ATP-binding protein	−1.99 (0.48)	−2.07 (p<0.05)

Data are from three biological replicates.

a Average n-fold change in expression for three biological and three technical replicates, each. Values in parentheses are standard deviations.

b n-fold change after expression array analysis of three biological replicates. Significance is indicated by p-values in parentheses.

## Discussion

Oral microbial diseases are based on qualitative and quantitative dysbalances of the physiological and pathogenic microbial flora and thus are polymicrobial by nature. Comparability of current *in vitro* investigations on microbial gene expression during health and diseased status is limited due to usage of diverse experimental setups. Differences include environmental conditions as well as static or flow through culture systems. The informative value of bacterial transcriptome studies is restricted in most cases due to the single-species DNA array setups. To overcome some of these shortcomings we here describe the setup and use of a 5-species transcriptome array for the study of both single- and multi-species cultures and biofilms. Only a minority between 0.35% (in case of *P. ginivalis*) and up to 1.15% (in case of *S. mutans*) of probes displayed unspecific hybridization results. Due to the high redundancy of probes per gene, these unspecific probes could be excluded from further analysis while the gene could still be included in the analysis in all but 3 cases.

In a first series of experiments, we simultaneously identified unspecific probes on the array and measured gene expression of early single-species planktonic cultures, monolayers and biofilms. Corresponding to the status of young biofilms, transcriptome analysis from the contained cells demonstrated the presence of transcripts of the vast majority of genes, especially such encoding all chaperones and ribosomal proteins, for example *dnaK* (SSA_2007; SMU_0082; PG_1208; AA000657) in *S. sanguinis*, *S. mutans*, *P. gingivalis* or *A. actinomycetemcomitans*, *clpL* (SSA_2096; PG_0010) in *S. sanguinis* and *P. gingivalis*, or a member of the *fts* operon, involved in cell division (SSA_0015; SMU_552; FN1451) in *S. sanguinis*, *S. mutans* and *F. nucleatum*. The ubiquitous presence of chaperone transcripts could be an indicator for general or acidic stress in these cultures [17], [25]–[27].

A complete *S. mitis* genome sequence was not available at the time of the array design, therefore the corresponding probes were not included. However, it was of special interest whether the array could still be used for a two-species analysis with a species represented on the chip and another that was not included. Again, specificity testing with *S. mitis* cDNA demonstrated cross hybridization of only a few *S. mutans* probes, allowing the inclusion of all *S. mutans* genes in the final analysis. The two-species combination was phenotypically described previously under growth conditions which supported planktonic growth of both species, but biofilm formation only for *S. mutans* [23]. The glucose-free growth medium CDM/sucrose is a prerequisite to mimick cariogenic biofilms with thick extracellular polysaccharides [28]. Under such virulent cariogenic conditions previous studies have shown suppression of *S. sanguinis* growth in co-culture with *S. mutans* [29].

Apparently, *S. mutans* suppressed *S. mitis* culturability in the biofilm, however, initial presence of *S. mitis* as a physiological species increased the cariogenic potential of the *S. mutans* biofilm. Does differential gene expression explain any of these phenotypes? For example, the co-cultivation of *S. mitis* with *S. mutans* resulted in high transcript abundances of *grpE* (SMU_81). However, only the inspection of the whole operon (SMU_80-83) allowed the determination that all genes of this operon were found in higher transcript abundances compared to the *S. mutans* mono-species culture. In general, results of transcriptome analysis of the *S. mitis*/*S. mutans* co-culture were in good agreement with the former obtained functional results [23]. Standar *et al.* showed that the co-cultivation of both species led to higher biofilm masses compared to the *S. mutans* mono-species culture, whereas pH was lower in the *S. mitis*/*S. mutans* combination. Transcriptome analysis revealed that several genes encoding stress proteins of *S. mutans* were upregulated under the co-cultivation conditions. Earlier studies indicated an association between expression of *S. mutans* stress proteins at a lower pH-value. For instance, acid stress/shock induced transcription of the molecular chaperons *dnaK* and *groEL* [25], [27], which agreed with our findings in transcriptome and RT-qPCR analysis. Both proteins play essential roles in cellular metabolism by assisting in the folding of newly synthesized or denatured proteins, as well as in the assembly, transport, and degradation of cellular proteins [30]. Furthermore, HrcA, the repressor of class I heat shock gene expression and encoded by the first gene of the *dnaK* operon of *S. mutans* (*hrcA-grpE-dnaK*) negatively regulates the *dnaK* and the *groE* operon [27]. In the present study the *hrcA* gene transcript was decreased (p = 0.058), consistent with the increased presence of the g*roE* and *dnaK* operon transcripts.

Also the *clp* – system was shown to play an important role in acid tolerance [17], [26]. The genome of *S. mutans* UA159 encodes orthologs of five Clp ATPases (ClpB, ClpC, ClpE, ClpL, and ClpX), and a single ClpP peptidase [17], [31]. A *clpL* knock-out mutant of *S. mutans* displayed a raised sensitivity against acid killing [26]. Other studies could demonstrate that a ClpL homologue interacts with the HSP70 system, which includes DnaK and DnaJ to form a chaperone complex [32]. Therefore, one could speculate that ClpL plays an important role in acid stress tolerance. Here we could measure increased *clpL* transcript abundance in the presence of a low pH in the *S. mitis*/*S. mutans* co-cultures.

In good agreement with previous studies on *S. mutans* acid stress behavior [33], our study of the *S. mitis*/*S. mutans* co-culture revealed the upregulation of *copY* and *copZ*. In addition, the *copYAZ* operon was shown to be required for a specific copper tolerance [34] and for biofilm detachment, a late stage of the biofilm developmental process. *CopZ* – defective cells displayed decreased biofilm detachment [35]. Therefore, the presently observed increased biofilm thickness could be another consequence of the raised *copYAZ* transcript abundance upon *S. mutans* exposure to *S. mitis* cells.

Of note, previous studies have identified the agmatine deiminase system of *S. mutans* upregulated under increased pH and general stress [24]. Although an decreased pH was measured and an increase in general stress was assumed in the *S. mitis*/*S. mutans* co-culture, the agmatine deiminase system was found downregulated in our study. If this is specifically due to the presence of *S. mitis* needs to be determined in future studies.

Since cross-feeding and metabolic cooperation are also appreciated mechanisms acting in biofilms [5], [6], it is possible that end products of *S. mitis* can be used by *S. mutans* to enhance biofilm growth. This could also explain the higher biofilm masses in two-species culture. Possibly, *S. mitis* produces alternative carbohydrates for *S. mutans* metabolism. The upregulation of the sorbitol/mannitol – specific PTS (SMU_308 – SMU_314) and the major lactose transporter (SMU_1491 and SMU_1492), which was observed in this study, was earlier described as specific sugar inducible by the presence of sorbitol/manitol and lactose/galactose. *ptsG* encoding for a putative glucose-specific PTS IIABC component, which was also more abundant in our study, was shown to be induced by several mono-, di- or tri-saccharides. However, all these sugar uptake systems are only upregulated in the presence of their substrate [36]. *S. mitis* may influence their upregulation by the production of these substrates thereby allowing increases in biofilm mass production by *S. mutans*.

Another explanation for the increased biofilm masses could be the activity of the *S. mutans* glucosyltransferases. Although none of the *gtf* genes revealed a differential transcript abundance in the mixed culture, a previous study has documented their critical role in increased exopolysaccharide production and biofilm formation in *S. mutans* mixed-cultures with *S. oralis* and *A. naeslundii* [37]. Of note, also these authors showed a dominance of *S. mutans* in the mixed-species setting.

In the present study *gbpD* message decreased with increasing biofilm mass. Recently, GbpD was found to be unique to the *Streptococcus mutans* group. This protein binds to dextrane and additionally, releases fatty acids from triglycerides. Therefore, it was suggested a lipid-linked surface carbohydrate could be its natural substrate and its function could be the release of fatty acids from lipoteichoic acid [38]. Also GbpD was demonstrated to be important for single-species biofilm formation. A *gbpD* mutant formed a thinner biofilm [39], which is in contrast to the present findings. This discrepancy could be due to a *gbpD* downregulation as a consequence of a looser mixed-species biofilm structure allowing better fluid circulation and therefore higher bacterial growth rates. However, this needs to be evaluated in a future study.

Comparing different technologies to generate results like described in this study one can conclude that DNA arrays still have a cost benefit compared to deep sequencing RNAseq technologies like Illumina and Ion Torrent platforms. For most small laboratories the latter techniques are only available on an outsource basis, whereas array hybridization can be performed in many labs and array scanners are available in most departments. One major advantage of the array described here is its potential feasibility to investigate *in vivo* plaque samples in a clinical setting. This is currently studied in our group and includes evaluation of the minimum RNA concentration needed to get meaningful results after array hybridization.

In summary, we have developed a 5-species transcriptome array based on NimbleGen™ 385 K array technology. We showed that the chosen array setup can reliably detect the transcript abundance of whole genomes in single-species experiments. The obtained results uncovered genes which are actively transcribed under the defined environmental and culture conditions. Additionally, none specifically hybridizing probes were identified. They can be excluded from further analysis in multi-species investigations based on the individual specificity parameters and requirements of the researcher, however, in most cases without losing the whole gene for the analysis. To our best knowledge, so far there is no array available for multi-species transcriptome analysis. Therefore, the newly designed chip could be a powerful tool to simultaneously investigate the transcriptome of more than one species from the oral environment.

## Materials and Methods

### Bacterial strains and culture conditions

The bacterial strains *Streptococcus mitis* ATCC 11843, *Streptococcus mutans* UA159, *S. sanguinis* SK36, *F. nucleatum* ATCC 25586, *P. gingivalis* W83 and *A. actinomycetemcomitans* HK1651 were purchased from commercial providers (DSMZ, Braunschweig, Germany and ATCC, Manassas, USA).

*Streptococcus mitis* ATCC 11843, *Streptococcus mutans* UA159, *S. sanguinis* SK36 and *A. actinomycetemcomitans* HK1651 were cultured in brain heart infusion medium (BHI; Oxoid) at 37°C under a 5% CO_2_–20% O_2_ atmosphere. *F. nucleatum* ATCC 25586 and *P. gingivalis* W83 were cultivated in BHI supplemented with 0.25% glutamate or 5 µg/ml hemin and 50 mM galactose, respectively, at 37°C under a 10% CO_2_ – 10% H_2_ – 80% N_2_ atmosphere.

### Setup of adherent cell cultures and biofilms

Bacteria were grown in BHI to stationary phase, washed with phosphate buffered saline (PBS, pH 7.4), and adjusted to a strain specific OD_600_ to obtain 1×10∧8 cells ml∧-1. Subsequently, each bacterial suspension was diluted 10-fold in a chemical defined medium (CDM) supplemented with 50 mM sucrose (*S. mutans*, *S. sanguinis*, *A. actinomycetemcomitans*) or in artificial saliva supplemented with 50 mM galactose (*P. gingivalis* and *F. nucleatum*) and inoculated in 75 cm^2^ CELLSTAR® tissue culture flasks (greiner bio-one, Frickenhausen, Germany). The bacteria were cultivated alone to establish species-dependent mono-species cultures and biofilms or in combination resulting in two-species biofilms. All cultures were grown in an anaerobic incubator under an appropriate atmosphere (80% N_2_, 10% CO_2_, 10% H_2_) at 37°C for one day under static conditions. The incubator atmosphere was saturated with water vapor to prevent exsiccation of the cultures and it was constantly exposed to a platinum catalyst to decrease the content of short-chained fatty acids in the atmosphere. Monolayer formation and biofilm growth was determined as described previously [23].

### RNA preparation from whole biofilm samples, cDNA synthesis, labeling and hybridization

To obtain samples for RNA preparation, mono- and two-species cultures and biofilms were set up in uncoated CELLSTAR® tissue culture flasks (75 cm^2^, 250 ml) (Greiner Bio-One, Frickenhausen, Germany) and allowed to build for one day of incubation under anaerobic conditions. In the cases when an isolate only formed a cell monolayer, the culture supernatant and the cells removed from the monolayers were combined to achieve a significant cell mass for RNA preparation (*A. actinomycetemcomitans*, *F. nucleatum*, *P.* gingivalis). For species with the capacity to form thick biofilms (*S. sanguinis*, *S. mutans*) under the chosen assay conditions, liquid medium was removed and biofilms were gently washed with PBS. Biofilms were detached from the surface by thorough scraping and washing with PBS. The biofilm suspension was centrifuged and the obtained pellet was used immediately or frozen at −80°C until further use.

RNA preparation was carried out from 100 mg cell material with the Fast RNA® Pro Blue Kit (MP Biomedicals, Solon, Ohio) following manufacturers instructions. To exclude DNA contaminations, the isolated and purified RNA was digested with DNase I for 1 hour at 37°C. Subsequently, the RNA was controlled for DNA contaminations by 16S rDNA PCR. Absence of amplified PCR products was confirmed by agarose gel electrophoresis. The RNA concentration was determined with the Picodrop photometer (Biozym, Oldendorf, Germany).

cDNA synthesis and labeling was done with the superscript II cDNA-Synthesis-Kit according to the manufacturer's instructions (Invitrogen, Karlsruhe, Germany). A total amount of 10 µg RNA was used for cDNA synthesis.

The labeling of the cDNA was done like recommended by the Nimble Chip Arrays User's Guide (Gene Expression Analysis v 2.0).

Hybridization of custom 385 K microarray slides with the prepared cDNA was carried out at 42°C overnight using a MAUI Hybridization System following the manusfacturer's instructions (BioMicroSystems, Salt Lake City, UT, USA). A stringent washing protocol of the arrays was performed following the instructions of the Nimble Chip Arrays User's Guide (Gene Expression Analysis v 2.0). The microarrays were stored dry and in the dark until the arrays were scanned.

### Chip specifications

The probe sets covering the 5 different bacterial genomes were designed by NimbleGen according to the company's protocols and algorithms, and all probes were manufactured directly onto the slides via photolithographic synthesis of 60 mer oligonucleotides®. The array probe sets are based on the following whole genome sequences deposited at NCBI: *F. nucleatum* ATCC 25586 (NCBI, full genome, accession no. AE009951) with 1964 genes, *P. gingivalis* W83 (NCBI, full genome, accession no. AE015924) with 1883 genes, *S. sanguinis* SK36 (NCBI, full genome, accession no. CP000387) with 2244 genes, *S. mutans* UA159 (NCBI, full genome, accession no. AE014133) with 1927 genes, and *A. actinomycetemcomitans* HK1651 (full genome available at: http://www.oralgen.lanl.gov/oralgen/downloads) with 2168 genes.

For every gene sequence up to 13 individual probes were designed to cover the gene sequence of interest (for detailed information refer to Supporting Tables S3, S5 and S6), resulting in 10.107 unique probe sets covering 10.186 genes. Each probe was synthesized in three replicates located in different array regions. This setup guaranteed maximum reproducibility of the hybridization process and fluorescence signals. In some cases the NimbleGene algorithms were unable to design adequate probes for certain genes. The genes not covered by the current design are listed in Supporting Table S7.

Additionally, there were 3510 random probes arbitrarily distributed on the chip, which served as negative controls. This entire setup allowed the identification of unspecific reactions without the need to exclude whole genes from the analysis in most cases. The decision if genes with cross-hybridizing probes are eliminated from the investigation could be based on the ratio of nonspecifically reacting probes in relation to the total number of probes for that particular gene. For this study here, we decided to exclude all *S. mutans* probes which cross-hybridized after *S. mitis* chip hybridization, but not whole genes from the analysis of the *S. mitis*/*S. mutans* two-species combination.

### Scanning and quality control of the microarrays and hybridization result interpretation

Scanning of microarrays and subsequent feature extraction was done with the DNA Microarray Scanner from Agilent (Agilent Technologies, Waldbronn, Germany, resolution: 5 µm) and NimbleScan™ software (version 2.4, NimbleGen, Madison, USA), respectively.

Each probe had to pass several criteria of quality control before entering the analysis tool GeneSpring GX (version 11.0, Agilent Technologies, Waldbronn, Germany). These criteria comprised:

- Identification of probe replicates influenced by wash artifacts: All *.pair data files resulting from scanning and feature extraction were analyzed for inhomogeneous replicates of every probe. Replicates with more than a two-fold aberration from the intensity of the other two replicates of this probe were excluded from analysis. In case of more than a two-fold aberration over all three replicates of a single probe, all replicates of this probe were deleted.
- Detection of unspecific inter-species cross-reactions: After using cDNAs from all species arranged on the chip for single-species hybridizations, all probes of the other species were checked for their potential hybridization signals. In case of a signal intensity exceeding that of the mean of all random probes on this array by a factor of two, these probes were designated as nonspecifically inter-species cross-reacting (for illustration please refer to supporting Figure S2a–e).
- Elimination of results from nonspecifically inter-species cross-reacting probes: When using the cDNAs from the two-species combination *S. mutans* and *S. mitis*, cDNA from the latter was checked for cross-hybridization with probes of *S. mutans* UA159 present on the chip. Employing the criteria from above, a probe was declared as false positive when the derived signal intensity after hybridization with *S. mitis* cDNA was more than two-fold higher than the mean of all random probes on this array, i. e. the negative control. Again, these nonspecific positive probes were excluded from analysis when comparing the *S. mutans* single-species transcriptome with the *S. mutans* transcriptome after co-culture with *S. mitis*.

Normalization and background correction was done by RMA [Robust Multi-Array Analysis, [40], [41]] with the NimbleScan Software summarizing all probes per gene resulting in single values per gene. The so obtained *.calls data files were analyzed with GeneSpring GX (version 11.0, Agilent Technologies).

All genes of a species, which passed the quality control inspection criteria, were finally grouped into 4 different categories of transcript abundance related to the maximal fluorescence signal intensity of the array (refer to supporting Table S1).

### RT-qPCR

Real time PCR (RT-qPCR) was performed for a subset of genes of *S. mutans* UA159, which were found to be up- or down-regulated in transcriptome analysis of *S. mutans* UA159 co-cultured with *S. mitis* compared to the *S. mutans* mono-species biofilm. Only genes leading to unequivocal hybridization results were used for primer design. Primers were designed with the help of the free online tool Primerfox (http://www.primerfox.com/, Supporting Table S8). For each set of primers, a standard amplification curve was plotted (critical threshold cycle against log of template DNA concentration) and all primers were experimentally controlled for primer dimer formation. RNA used for cDNA synthesis was controlled for DNA contamination as described earlier in this methods section. cDNA synthesis was done with the superscript II cDNA-synthesis-kit from Invitrogen accordant to manufacturer's instructions.

The RT-qPCR reaction mixture (25 µl) contained 1× MAXIMA Sybr Green Master Mix (Fermentas), 6.5 µl cDNA sample and 1 mM of the appropriate forward and reverse PCR primers (Table S3). RT-qPCR reactions were done in 96 well optical reaction plates with the ABI PRISM® 7000 Sequence Detection System (Applied Biosysthems). Conditions of RT-qPCR were as follows: 2 min 50°C, 10 min 95°C, 15 sec 95°C, 1 min 60°C. The last two steps were repeated 40 times.

Each assay was performed with at least three biologically independent RNA samples in technical duplicates.

The critical threshold cycle (C_t_) was defined as the cycle in which fluorescence becomes detectable above the background fluorescence and is inversely proportional to the logarithm of the initial number of template molecules. Relative quantification was performed with the comparative C_t_ method, also known as the 2^−[delta][delta]Ct^ method. C_t_ values for the genes of interest were normalized to the gene SMU_2015.

### Reproducibility and statistics

Each experiment was performed on at least 3 independent occasions (biological replicates) with 2 or 3 replicates each (technical replicates). Statistical parameters (mean, median, standard deviation, p-values) were determined employing the Windows Excel program, and GenSpringGX. P-values less than 0.05 were considered as significant.

### Accession number

The raw data and meta information on the experiments have been deposited in the Gene Expression Omnibus database (GEO; www.ncbi.nlm.nih.gov/geo/) and received the accession number GSE28841.

## Supporting Information

Figure S1 Documentation of biofilm and monolayer formation of single-species cultures of *A. actinomycetemcomitans; F. nucleatum*, *P.* gingivalis, *S. sanguinis*, and *S. mutans*.
(PDF)

Figure S2 Histogram of array fluorescence intensities.
(PDF)

Table S1 Graduated fluorescence intensities of genes passing the quality control criteria.
(XLS)

Table S2 Data set of crosshybridizing probes and the corresponding genes.
(XLS)

Table S3 All genes represented on the array, including their appropriate probe number.
(XLS)

Table S4 All probes and their corresponding genes which were found as biological false positives after hybridization with RNA of *S. mitis.*
(XLS)

Table S5 All genes represented on the array, with less than 13 probes.
(XLS)

Table S6 Genes, which share probes with other genes.
(XLS)

Table S7 All genes for which NimbleGen algorhithms were not able to identify adequate probes.
(XLS)

Table S8 Data set of RT-PCR primer.
(PDF)
