# Supplementary material for: A Five-Species Transcriptome Array for Oral Mixed-Biofilm Studies
Source: PLoS One. 2011 Dec 14;6(12):e27827. doi: 10.1371/journal.pone.0027827 (PMC3237422; doi:10.1371/journal.pone.0027827)
Supplement: Figure S1 — Documentation of biofilm and monolayer formation of single-species cultures of A. actinomycetemcomitans; F. nucleatum, P. gingivalis, S. sanguinis, and S. mutans. (PDF) [file pone.0027827.s001.pdf]

Supporting Information of "A five-species transcriptome array for oral mixed-biofilm studies“; Redanz *et al*. 2011;

Figure S1

A) *S. sanguinis*

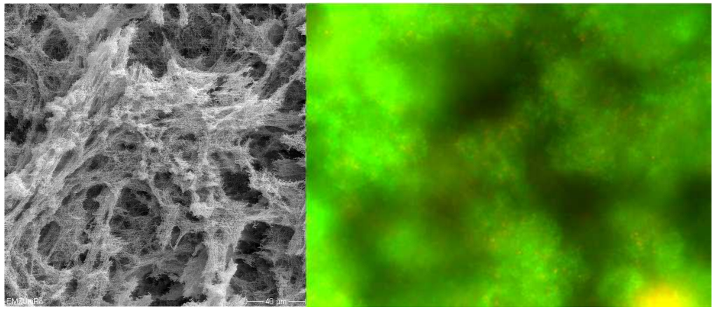

B) *S. mutans*

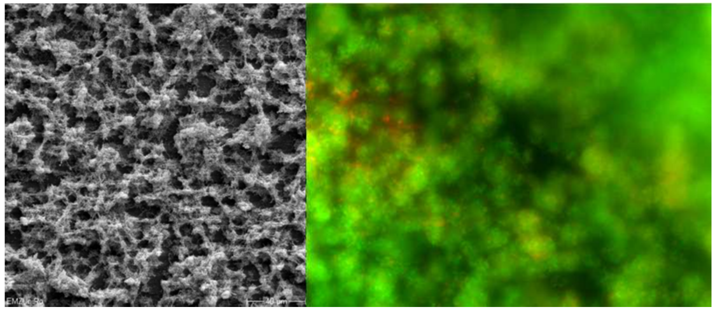

C) *P. gingivalis*

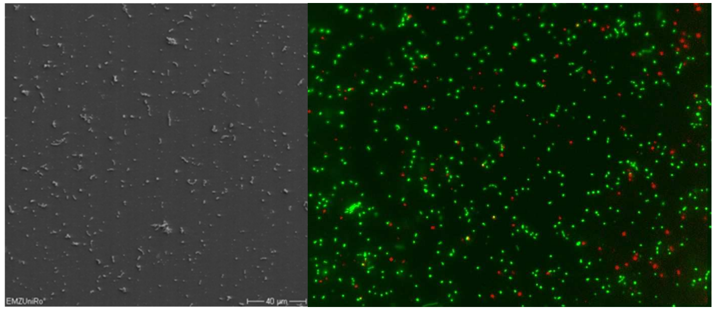

C) *F. nucleatum*

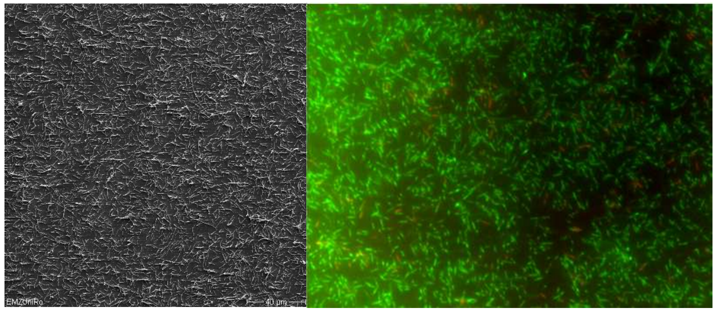

C) *A. actinomycetemcomitans*

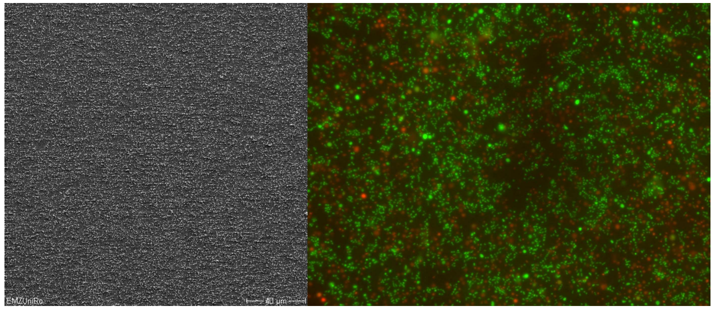

Shown are scanning electron (SEM) and fluorescence microscopic illustrations of the mentioned species. For SEM, bacterial cells were cultured anaerobically in CDM / succrose using uncoated polystyrene 24-well-plates (Greiner Bio-One), each well containing a uncoated, sterile plastic coverslip (13mm diameter; Nunc, Wiesbaden, Germany). After 24 h, biofilms on coverslips were fixed for 24 h in 2.5 % glutardialdehyde, subsequently washed with 0.1 M Na-acetate buffer (pH 7.3) and dehydrated in a graded series of ethanol. Coverslips were subjected to critical point drying with CO_2, sputter-coated with gold (thickness approx. 10 nm), and examined with a Zeiss DSM 960A electron microscope. For fluorescence microscopy cells were cultured equally, but stained with BacLight Live / Dead (Molecular Probes, Eugene, Oregon) and inspected by fluorescence microscopy (BX60 microscope, Olympus, Hamburg, Germany).
