# Supplementary material for: A Five-Species Transcriptome Array for Oral Mixed-Biofilm Studies
Source: PLoS One. 2011 Dec 14;6(12):e27827. doi: 10.1371/journal.pone.0027827 (PMC3237422; doi:10.1371/journal.pone.0027827)
Supplement: Figure S2 — Histogram of array fluorescence intensities. (PDF) [file pone.0027827.s002.pdf]

Supporting Information of "A five-species transcriptome array for oral mixed-biofilm studies"; Redanz *et al*. 2011;

Figure S2

A

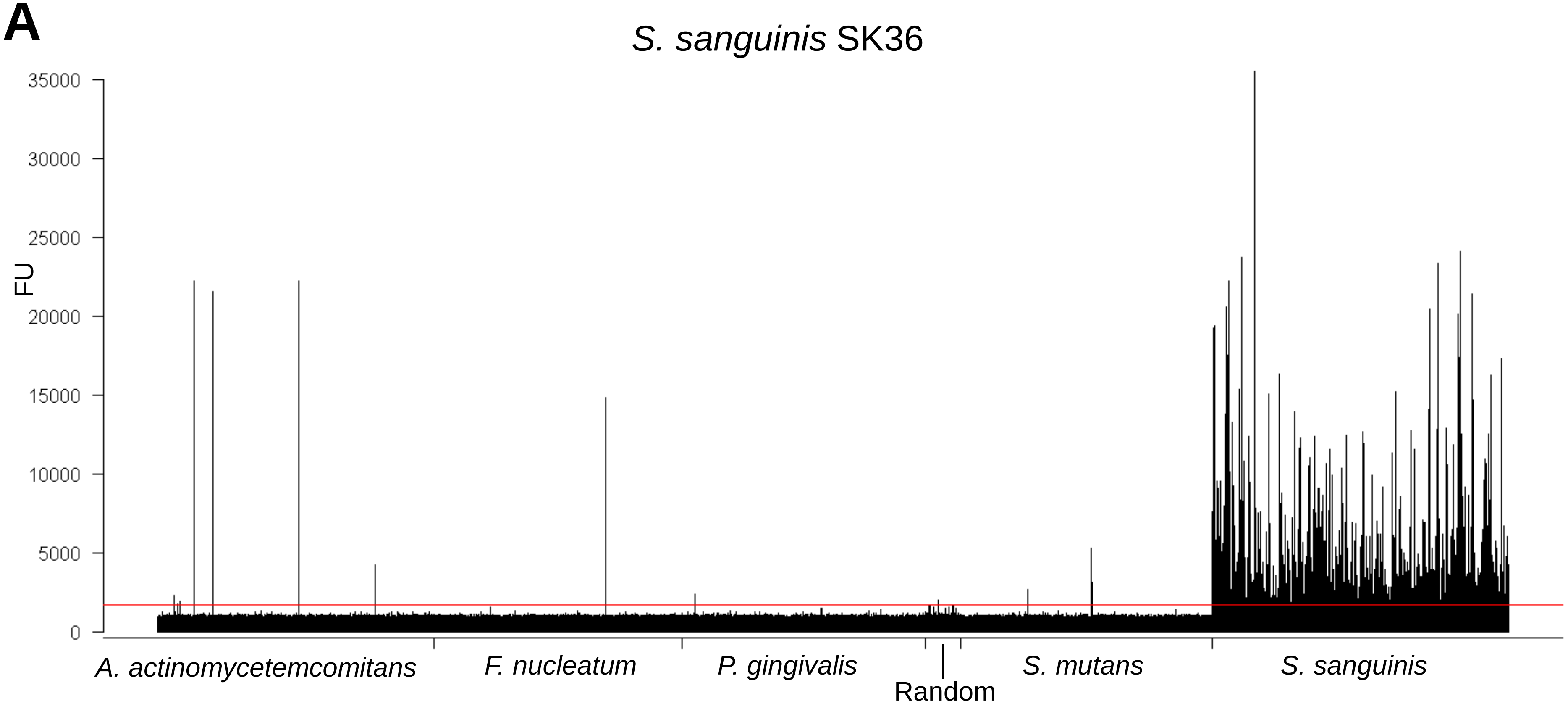

B

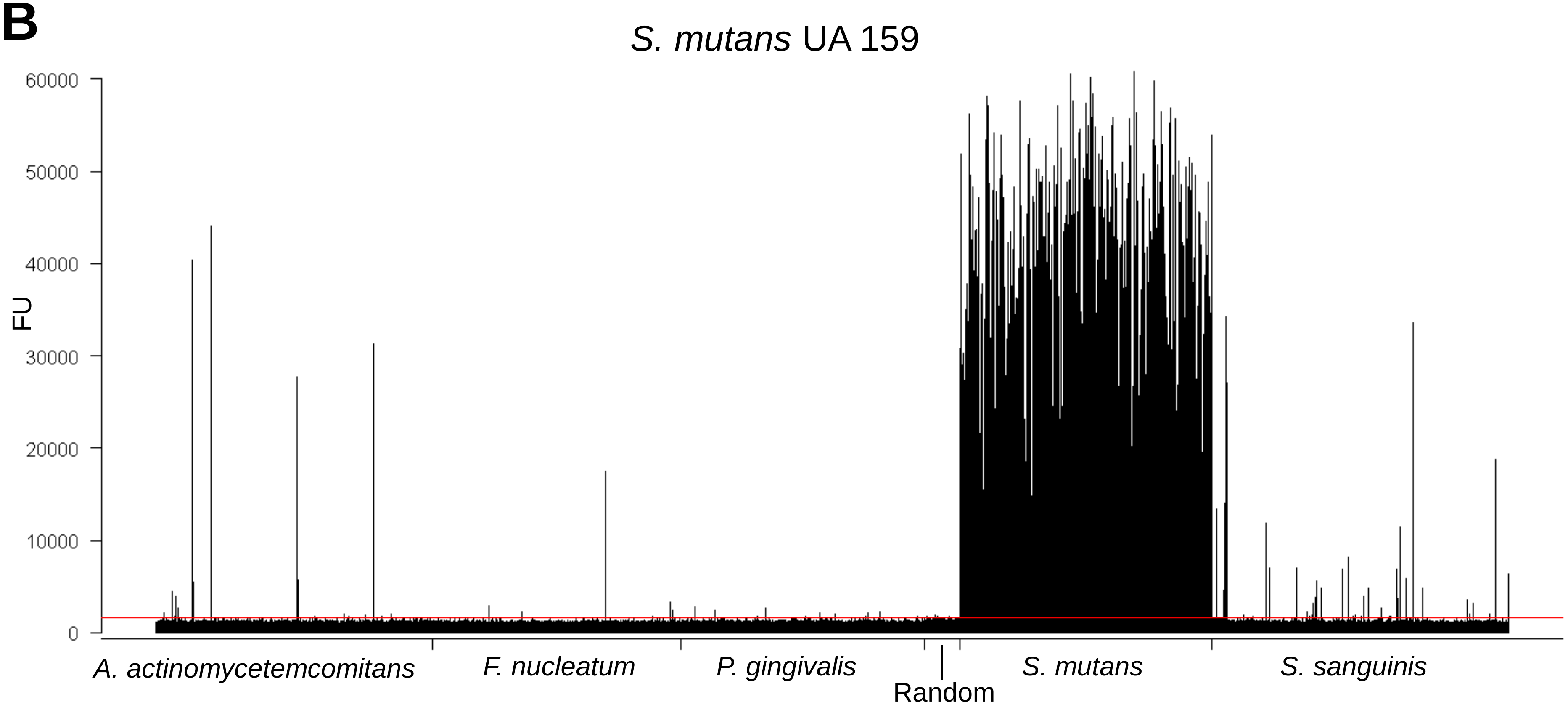
S. mutans UA 159
60000
50000
40000
30000
20000
10000
0
FU
A. actinomycetemcomitans
F. nucleatum
P. gingivalis
Random
S. mutans
S. sanguinis

C

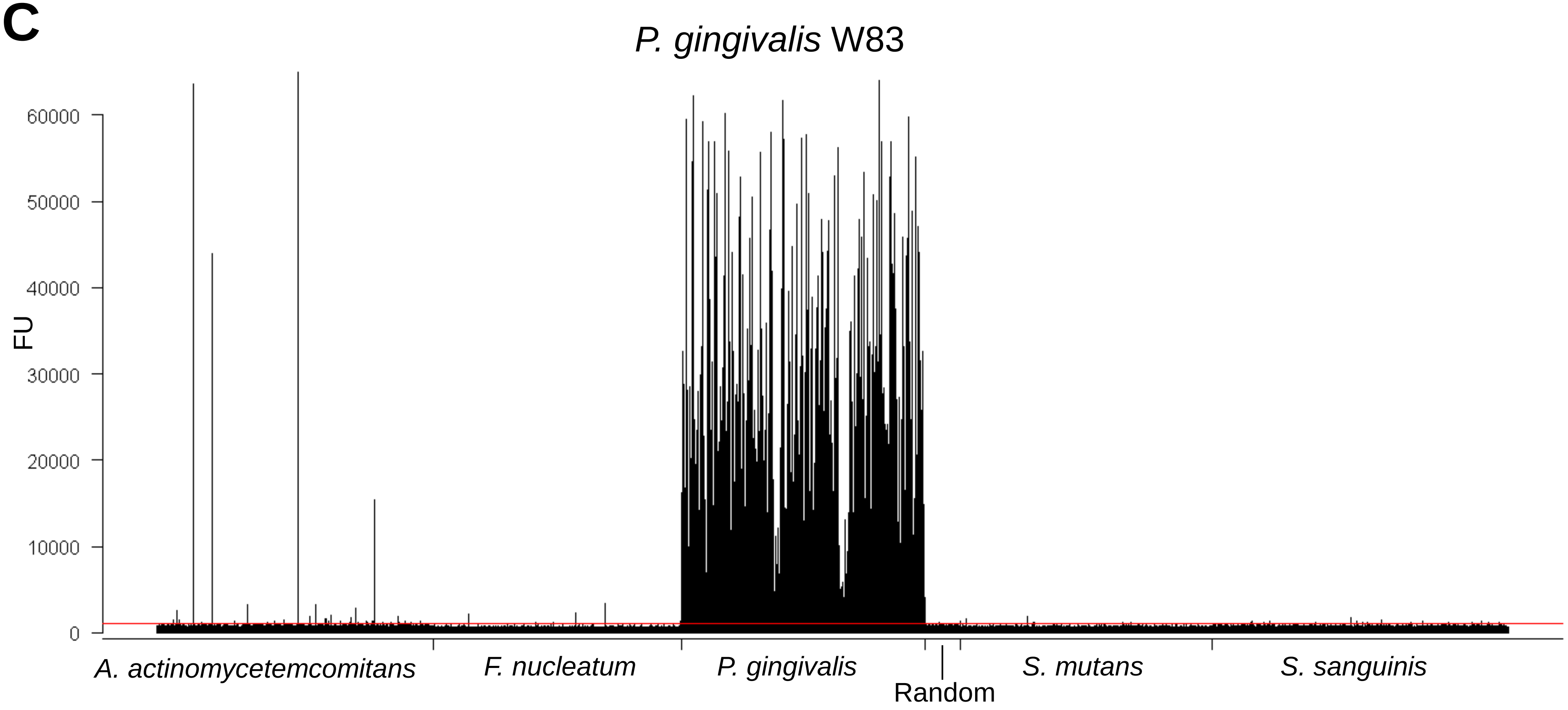

P. gingivalis W83
FU
60000
50000
40000
30000
20000
10000
0
A. actinomycetemcomitans
F. nucleatum
P. gingivalis
Random
S. mutans
S. sanguinis

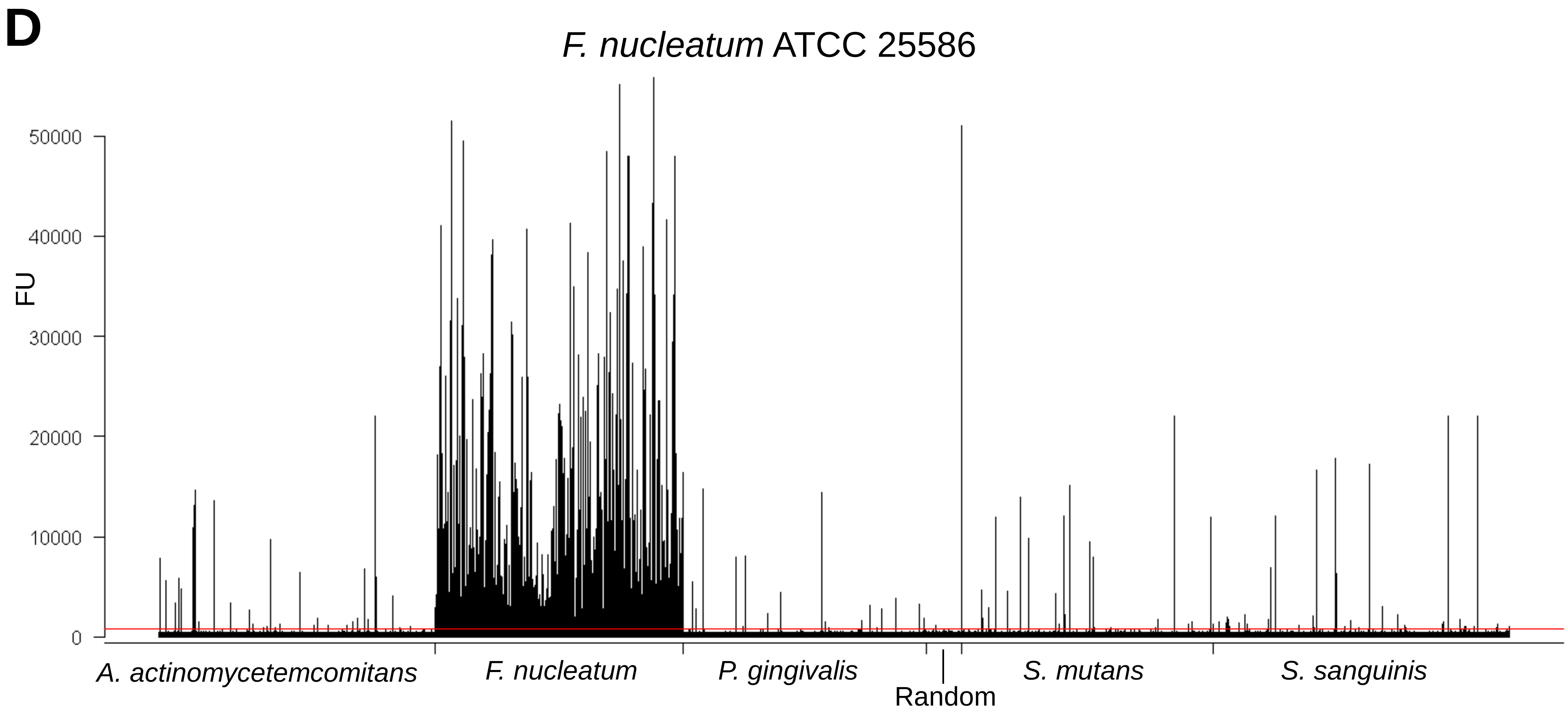

D
F. nucleatum ATCC 25586
FU
50000
40000
30000
20000
10000
0
A. actinomycetemcomitans
F. nucleatum
P. gingivalis
Random
S. mutans
S. sanguinis

E

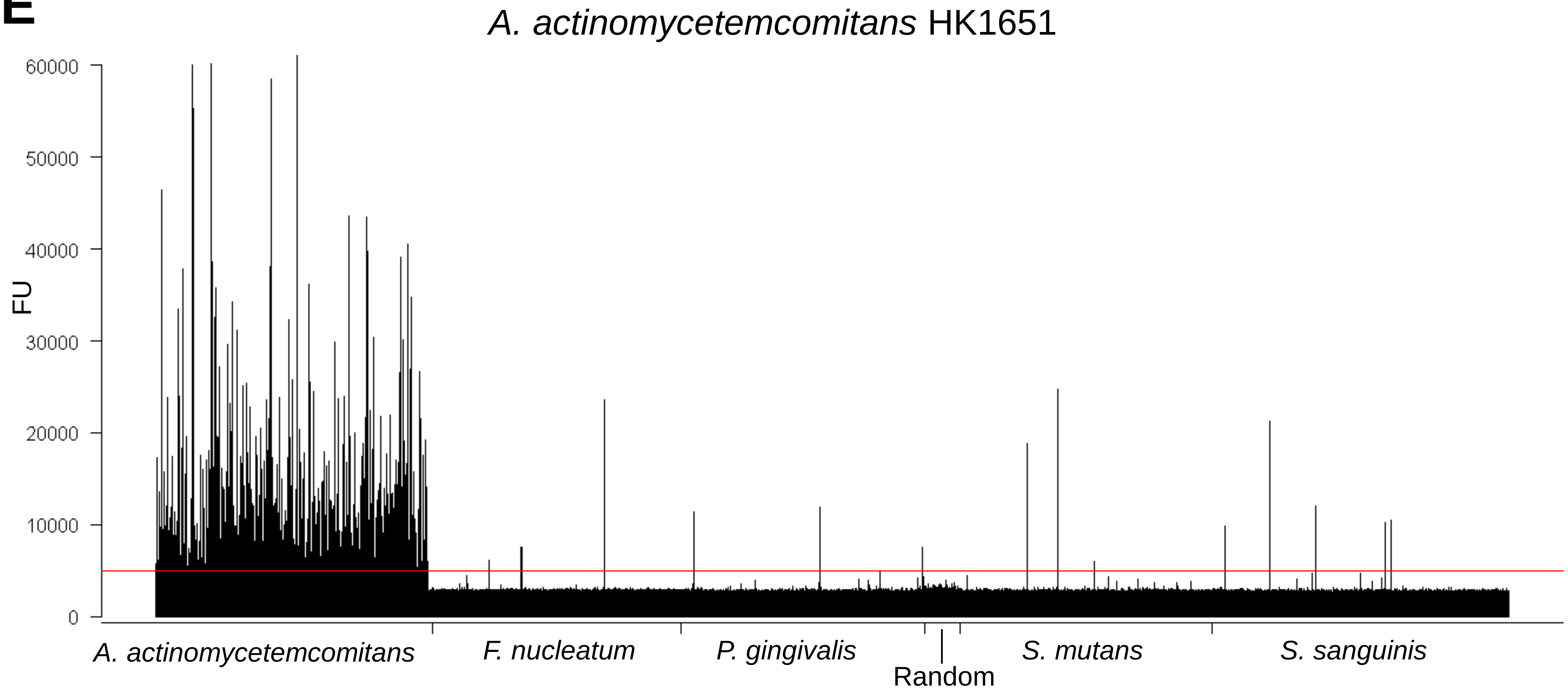
A. actinomycetemcomitans HK1651
60000
50000
40000
30000
20000
10000
0
FU
A. actinomycetemcomitans
F. nucleatum
P. gingivalis
Random
S. mutans
S. sanguinis

Supporting information Figure S2: Shown are the fluorescence intensities of all probes of all species represented on the array described in this study. The fluorescence intensities of all technical probe replicates were averaged after technical false positive correction.
A: Hybridization with *S. sanguinis* SK36; B*: S. mutans* UA 159; C: *P. gingivalis* W83; D: *F. nucleatum* ATCC 25586; and E: *A. actinomycetemcomitans* HK1651. The red line marks the cutoff value for the declaration of positive signals (2*random-mean, each).
