# Supplementary material for: A Five-Species Transcriptome Array for Oral Mixed-Biofilm Studies
Source: PLoS One. 2011 Dec 14;6(12):e27827. doi: 10.1371/journal.pone.0027827 (PMC3237422; doi:10.1371/journal.pone.0027827)
Supplement: Table S8 — Data set of RT-PCR primer. (PDF) [file pone.0027827.s010.pdf]

Supporting Information of "A five-species transcriptome array for oral mixed-biofilm studies"; Redanz *et al*. 2011

Table S8: Primer designed for RT-PCR.

**primer name**	**sequence 5`- 3`**	**gene info**
RT-PCR primer for the gene with no change of expression		
smu.2015_F:	GGTGATGCTGTGTCAAATGC	rplE, 50S ribosomal protein L5
smu.2015_R:	CCGGCGATTGATTTCTTAGC	
RT-PCR primer for upregulated genes		
smu.81_F:	GTTGCAAGAAGCACTTGAGC	grpE, heat shock protein GrpE
smu.81_R:	ACTTTGTCGCTCTTCATTAGC	
smu.312_F:	CTATTGTTGACTGTGGTGGC	PTS system, sorbitol phosphotransferase enzyme IIBC
smu.312_R:	TGTATTGGGCTAAAGGTCCG	
smu.426_F:	AAAGGTCGAACCTCAGATGC	copA, copper-transporting ATPase; P-type ATPase
smu.426_R:	GCACTTGCTCAATAGGAACC	
smu.956_F:	ACTGCTGAGATCTTAGCTCG	clp, putative Clp-like ATP-dependent protease, ATP-binding subunit
smu.956_R:	TAACAATAGCCTGTGCTAAGC	
smu.1495_F:	GGGACCTATGACTTTACACG	lacB, galactose-6-phosphate isomerase subunit LacB
smu.1495_R:	GTACCGCACATACATACACC	
smu.1954_F:	GCTAACCCAATCGGTATTCG	groEL, chaperonin GroEL
smu.1954_R:	GCTTCTTTGCCAGAAACAGG	
smu.2047_R:	TGTCCTACCTTGAAACGAGC	ptsG, putative PTS system, glucose-specific II ABC component
smu.2047_F:	ACTTTGGTTAGCTTGGGTGC	
RT-PCR primer for downregulated genes		
smu.262_F:	CAAACCTCAACACGAACACG	Otc, putative ornithin carbamoyltransferase
smu.262_R:	TCGTTTCATGACCTCCAAGC	
smu.498_F:	GGATCTATCTACTGTCGAGC	comF, putative late competence protein
smu.498_R:	CTTAAGACCTGACCCTTTGG	
smu.772_F:	CTACTGAAGCAGAGAAGACC	gbpD, putative glucan-binding protein D, BglB-like protein
smu.772_R:	AACATTTCTACCATCAGCACC	

smu.1055_R:	AAATCGCAGGTCATTCTCGC	radC, putative DNA repair protein RadC
smu.1055_F:	AGACGATCTGTTGCTGAACC	
smu.1340_F:	ATACAGAAGTTATGCTACATGG	bacA2, putative surfactin synthetase
smu.1340_R:	TATCAATGATAGATAGGCATGC	
smu.1934c_R:	CTGCCAAACTGACTCTTTGC	putative cobalt ABC transporter, ABC-binding protein
smu.1934c_F:	GACAATGTTTGAGAGGGTGG	
